# Expression profile and prognostic value of *CXCR* family members in head and neck squamous cell carcinoma

**DOI:** 10.1186/s12957-022-02713-z

**Published:** 2022-08-17

**Authors:** Yiming Shen, Chongchang Zhou, Yujie Cao, Qun Li, Hongxia Deng, Shanshan Gu, Yidong Wu, Zhisen Shen

**Affiliations:** 1 Department of Otolaryngology, Head and Neck Surgery, The Affiliated Lihuili Hospital, Ningbo University, Ningbo, China; 2 Department of Otolaryngology, Head and Neck Surgery, Ningbo Medical Center Lihuili Hospital, Ningbo, China; 3 Medical School of Ningbo University, Ningbo, 315000 China

**Keywords:** Head and neck squamous cell carcinoma, CXC chemokine receptor gene family, The Cancer Genome Atlas, Prognosis, Survival

## Abstract

**Background:**

CXC chemokine receptor gene family consists of seven well-established members which are broadly involved in biological functions of various cancers. Currently, limited studies have shed light on the expression profile of CXCR family members (*CXCRs*), as well as their prognostic value, in head and neck squamous cells carcinoma (HNSCC).

**Methods:**

The data for this study were retrieved from the Cancer Genome Atlas database and other publicly available databases, including gene expression, methylation profiles, clinical information, immunological features, and prognoses. The expression pattern and prognostic values of *CXCRs* were identified, and the potential mechanism underlying *CXCRs* function in HNSCC was investigated by gene set enrichment analysis (GSEA).

**Results:**

*CXCRs* were differentially expressed in HNSCC. As shown by Kaplan–Meier analysis, high *CXCR3-6* expression was significantly associated with better prognostic outcomes of HNSCC patients, including overall survival and progression-free survival. According to the results of univariate and multivariate Cox proportional risk regression analysis, it was demonstrated that upregulation of *CXCR3-6* was an independent factor for better prognosis, while the two other clinical features, age and stage, were factors for worse prognosis. A significant positive correlation between *CXCR3-6* and tumor-infiltrated immune cells was revealed by results from Tumor Immune Estimation Resource and CIBERSORT analysis database. The main involvement of *CXCRs* in immune and inflammatory responses was further confirmed by GSEA.

**Conclusions:**

Overall, this study provided a rationale for targeting *CXCRs* as a promising therapeutic strategy of HNSCC.

**Supplementary Information:**

The online version contains supplementary material available at 10.1186/s12957-022-02713-z.

## Background

Head and neck squamous carcinoma (HNSCC) is the most common pathological subtype of head and neck cancer which is a highly aggressive malignancy [1]. The annual number of people diagnosed with head and neck cancer worldwide has reached nearly 500,000, and the mortality rate of the patients diagnosed with HNSCC has increased over the last few years [2]. Although prominent progress has been made in the treatment of HNSCC, the 5-year survival rate of HNSCC patients remains around 50% [3]. Therefore, further understanding of the complex cancer biology underlying this disease and developing more reliable predictive biomarkers for HNSCC prognosis are critical for facilitating the therapeutic efficacy of individualized treatment, which may ultimately help to enhance patients’ survival.

With more than 50 members, vertebrate G protein-coupled chemokine receptors are comprised of four different subfamilies, including CC chemokine receptors, XC chemokine receptors, CXC chemokine receptors, and CX3C chemokine receptors. CXC chemokine receptors are a large subfamily of chemokine receptors, which consists of seven members known as *CXCR1* to *CXCR7* [4]. *CXCR* family members (*CXCRs*) are well-known to participate in both anti-tumor immune response and tumorigenesis [5]. CXC chemokine receptors 1 and 2 (*CXCR1* and *CXCR2*), members of the receptors of ELR^+^ chemokines, are generally expressed simultaneously on neutrophils, fibroblasts, and vascular endothelial cells and have a relation to the activation of multiple downstream signaling pathways. A high affinity exhibited by both *CXCR1* and *CXCR2* has been observed toward the CXC motif chemokine ligand (*CXCL*) 8, a common ligand secreted by cancer cells and inflammatory cells. Binding of the ligand to these receptors promotes tumor invasion, angiogenesis, and metastasis [6, 7]. *CXCR3-6* are the receptors of the ELR^−^ chemokines [8]. *CXCR3* can bind to *CXCL9*, *CXCL10*, and *CXCL11*, respectively, and its expression can be detected on various subtypes of natural killer (NK) cells and T cells [9–12]. *CXCR3* exhibits diverse biological functions upon extracellular stimuli which activate intracellular signal transduction pathways that involved in survival, cell behavior, and cell identity. *CXCR4*, an important regulator of the homing and mobilization of hematopoietic cell, is widely expressed on different cell types like megakaryocytes, embryonic stem cells, vascular endothelial cells, and cancer cells [13, 14]. The interaction of *CXCR4* with its ligand, *CXCL12*, has been shown to facilitate the migration of T and B lymphocyte. The *CXCR5* is a receptor for *CXCL13* which is a homeostatic chemokine. Upon contact with *CXCL13*, *CXCR5* is capable of regulating cell migration [15]. The overexpression of *CXCR5* and its cognate ligand *CXCL13* has been implicated in many different types of cancer [16]. Expressed on CD8^+^ T, NK, and CD4^+^ T cells, C*XCR6* could promote the recruitment of tumor-infiltrating lymphocytes in combination with *CXCL16* expressed by tumor cells, thus contributing to a better prognosis for cancer patients [17]. *CXCR7* is previously known as *ACKR3*. Although it is also a seven-transmembrane receptor, *CXCR7* cannot mediate the classical Gi signaling pathway like *CXCR3* and many other chemokine receptors [18]. *CXCR7* has previously been regarded as a “decoy” and an atypical chemokine receptor for *CXCL12*. Moreover, *CXCR7* is a promising therapeutic target with its involvement in tumorigenesis and autoimmune disorders. However, the mechanism by which *CXCRs* are activated or deactivated in the development and progression of HNSCC remains unclear until now.

Here, we comprehensively analyzed the expression of the whole CXC chemokine receptor gene family and the potential correlation with prognostic value and clinical significance in HNSCC via different databases, including ONCOMINE and the Cancer Genome Atlas (TCGA), which provide detailed information about cancers. In addition, Tumor Immune Estimation Resource (TIMER) was employed to investigate the association between *CXCRs* and immune infiltration degree. To determine HNSCC-related risk factors, univariate/multivariate Cox regression models were utilized, and GSEA about *CXCRs*-related signaling pathways was performed.

## Methods

### Data resources

HNSCC transcriptomic sequencing data of 502 HNSCC and 44 normal samples were acquired from TCGA data (https://portal.gdc.cancer.gov/) [19]. The clinical features from the extracted clinical information of patients were included in the present study, including gender, age, stage, and tumor grade. Subsequently, the high-throughput sequencing (HTSeq) fragments per kilobase of transcript per million mapped reads (FPKM) data of *CXCRs* were download by utilizing Genomic Data Commons (GDC) Data Transfer Tool on the TCGA database. Besides, we also downloaded two HNSCC datasets (GSE41613 and GSE65858) from Gene Expression Omnibus (GEO) database to validate the association between *CXCR* members and overall survival. The basic characteristics of the HNSCC patients used in research are shown in Supplementary Table 1.

### Expression data of CXCRs in HNSCC

Gene expression of *CXCRs* was analyzed across multiple datasets by comparing tumor tissues to the healthy tissues, using ONCOMINE (http://www.oncomine.org) [20]. And the thresholds were set as follows: gene rank was set in the top 10%, data type was limited to mRNA, *p*-value was set to 0.01, and folding change (FC) was defined as 1.5. Based on the HTSeq FPKM data, Perl 5.26 software was adopted to extract the mRNA expression levels of *CXCR*s in HNSCC. The *limma* package in R software was employed to analyze how *CXCR*s were expressed differentially in HNSCC tissues and normal tissues. The *pheatmap* and *ggpubr* packages were utilized for the visualization of a heatmap and box plots to display analysis results.

### Relationship between methylation and mRNA expression of CXCRs in HNSCC

Data on the methylation levels of cg sites, which were within the gene promoter regions of differentially expressed *CXCRs* in HNSCC tissues, was downloaded from Illumina Human Methylation 450 K through GDC Data Transfer Tool on the TCGA and then was annotated with the annotation file from the official Illumina website (https://support.illumina.com/downloads/~infinium_humanmethylation450_product_files.html). After the methylation levels of cg sites were identified in *CXCRs* gene promoter regions, the *corrplot* package was utilized to understand how the methylation level and *CXCR* expression in HNSCC correlated.

### Prognostic value evaluation of CXCRs in HNSCC

The Kaplan–Meier method was adopted for the analysis of the survival rate. R package survival analysis was implemented combined with gene expression profiles and survival information of HNSCC involving a total of 499 HNSCC patients, to evaluate how the mRNA expression of *CXCR1-7* correlated with the prognoses of HNSCC patients. Then, the progression-free survival (PFS) was assessed along with overall survival (OS) time in search of the best optimal cutoff value. Moreover, for the estimation of the independent prognostic factors, we utilized univariate and multivariate Cox proportional hazards models. Forest plots were created to present the analysis results by using the *ggplot* package.

### Pearson correlation and protein-protein interaction (PPI) network of CXCRs

To determine the correlation among *CXCRs*, a Pearson correlation analysis was carried out using the *corrplot* package of R software genes on the basis of the gene expression data obtained from the TCGA database. In order to retrieve interacting genes or proteins, we established the PPI networks of *CXCRs* by the search tool (STRING, http:// www.string-db.org/).

### Gene set enrichment analysis (GSEA)

To further investigate how *CXCRs* participated in HNSCC carcinogenesis, GSEA was carried out (version 4.0.1; http://software. broadinstitute.org/gsea/index.jsp) to identify the *CXCRs*-associated signaling pathways in the TCGA HNSCC tissues [21, 22]. The identification of significant enrichment pathways employed a false discovery rate (FDR) of < 0.05 and a random combination number of 1000 for each mutation, with the reference to the annotated gene set file from the Msig database (c2.cp.kegg.v7.0.symbols.gmt).

### Corrections between tumor immune-infiltrating cells (TIICs) and CXCRs

During cancer progression, tumor cells interact with TIICs through a variety of genes and pathways. To explore the correlation of TIICs with *CXCRs*, the TIMER platform was adopted for analysis (https://cistrome.shinyapps.io/timer/), which is an online database incorporating expression profiles of over 10,000 samples across 23 cancer types from TCGA [23, 24]. According to TIMER, macrophages, CD4+ T cells, neutrophils, dendritic cells, B cells, and CD8+ T cells are the main components of the TIICs. The purity-corrected partial Spearman’s correlation (partial-cor) provided by TIMER was presented together with *p*-value in scatterplots in the current study. On the basis of the median value of gene expression, we divided the samples into high expression group and low expression group. The *vioplot* package of R software was utilized to visualize the analysis results. In a mixed cell population, the specific proportions that 22 tumor-infiltrating lymphocyte subsets accounted for respectively were estimated by the analytical tool CIBERSORT with gene expression data (https://cibersort.stanford.edu/).

## Results

### Differential expression of CXCRs in HNSCC patients

**Fig. 1:**
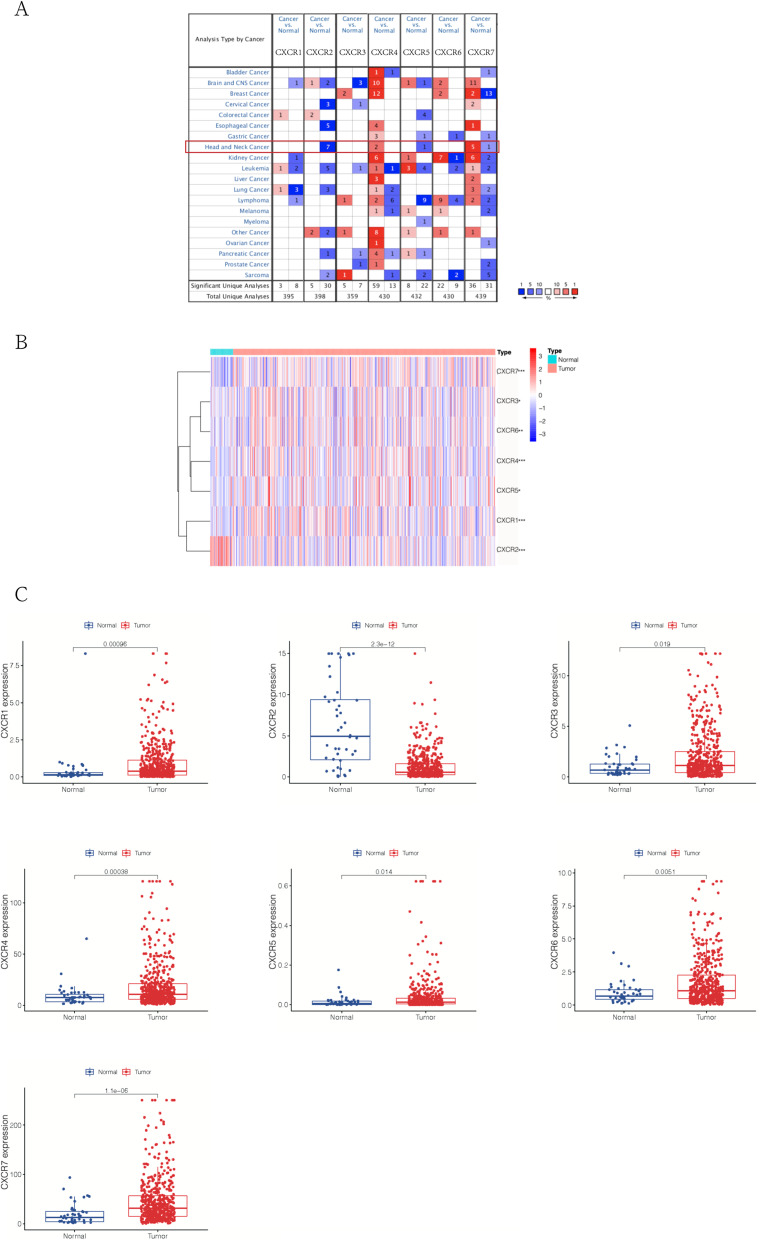
Expression of *CXCRs* in different types of cancers. **A** Red and blue in the heatmap indicate the numbers of datasets with increased and decreased levels of *CXCR* family members, respectively (*p* < 0.05). **B** Differential expression of *CXCRs* in HNSCC samples and normal tissues represented by a heatmap. The tree diagram on the left side of heatmap showed the cluster analysis between *CXCR* family members. *p < 0.05; **p < 0.01; ***p < 0.001. **C** Differential expression of *CXCRs* in HNSCC samples and normal tissues represented by box plots

It has been implicated that the aberrant expression of *CXCRs* exists in various cancer types like leukemia, lymphomas, and lung cancer. To assess the expression pattern of *CXCRs* in HNSCC, the gene expression data from ONCOMINE was analyzed. ONCOMINE analysis results shown in Fig. 1A demonstrated that mRNA expression levels of *CXCR4* and *CXCR7* were upregulated in all subtypes of head and neck cancer, while *CXCR2* and *CXCR5* were downregulated (fold change > 1.5, *p*-value < 0.05). And the mRNA expression level of *CXCR4* was notably upregulated in HNSCC tissue when compared with the healthy tissue (Table 1). In addition, 1.550-fold increase (*p*-value = 1.28E-10) and 2.378-fold increase (*p*-value = 1.49E-4) in the *CXCR6* expression in HNSCC tissues were found in two other studies [25, 26]. For the further understanding of the expression profile of *CXCRs* in HNSCC, based on 502 HNSCC samples and 44 healthy control samples from the TCGA database, Perl software and the *limma* package were utilized to obtain mRNA expression data of CXCR members (CXCR1–7) and analyze the differentially expressed *CXCRs.* The differential gene expression of *CXCRs* in the HNSCC samples, as well as normal tissues, was displayed in the heatmap generated by the *pheatmap* package (Fig. 1B). Furthermore, the aberrant expression levels of all *CXCR* members in HNSCC compared to the healthy tissues were revealed by the analysis results, especially the significant downregulation of *CXCR2* in HNSCC tissues, while the other *CXCRs*, namely *CXCR1* and *CXCR3* to *CXCR7*, were significantly upregulated in HNSCC tissues (Fig. 1C).

**Table 1:** Transcriptional levels of *CXCR* family members between normal tissues and HNSCC (ONCOMINE)

No.	Gene name	Fold change	*p*-value	*t*-test	References
1	*CXCR4*	3.447	2.75E-13	10.536	[20]
2	*CXCR4*	2.150	4.68E-6	5.002	[21]
3	*CXCR7*	2.185	3.24E-7	5.892	[20]
4	*CXCR7*	6.647	0.002	4.459	[22]
5	*CXCR7*	3.175	7.57E-4	3.497	[23]
6	*CXCR7*	1.550	1.28E-10	7.522	[21]
7	*CXCR7*	2.378	1.49E-4	3.937	[24]

### Methylation of the promoter regions of CXCR genes in HNSCC

**Fig. 2:**
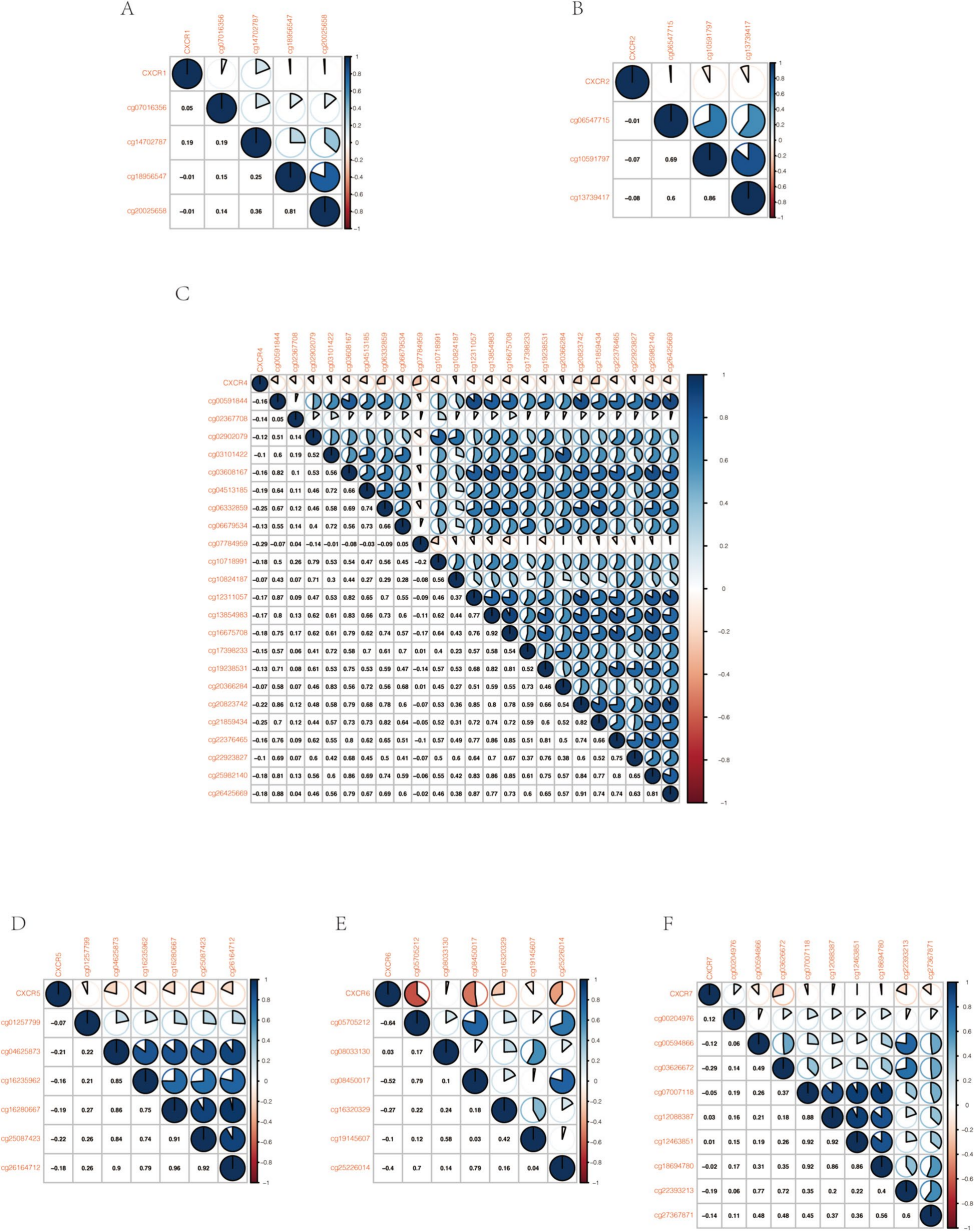
Correlation between methylation levels and expression of *CXCRs* in HNSCC

In the progression of cancer, one of the most typical mechanisms having a critical impact on gene expression is methylation of gene promoter regions. As Pearson’s correlation analysis indicated, among the identified seven *CXCR* members in HNSCC, five of the *CXCR* members, including *CXCR2* and *CXCR4* to *CXCR7*, were negatively correlated with methylation level (Fig. 2 B–F). Moreover, of the 4 evaluated cg sites in the *CXCR1* promoter region, only 2 were negatively associated with the expression of *CXCR1* in HNSCC (Fig. 2A). Together, the expression of *CXCR* members was inversely correlated with their methylation level in HNSCC according to the existing data.

### Prognostic value of CXCRs in HNSCC

**Fig. 3:**
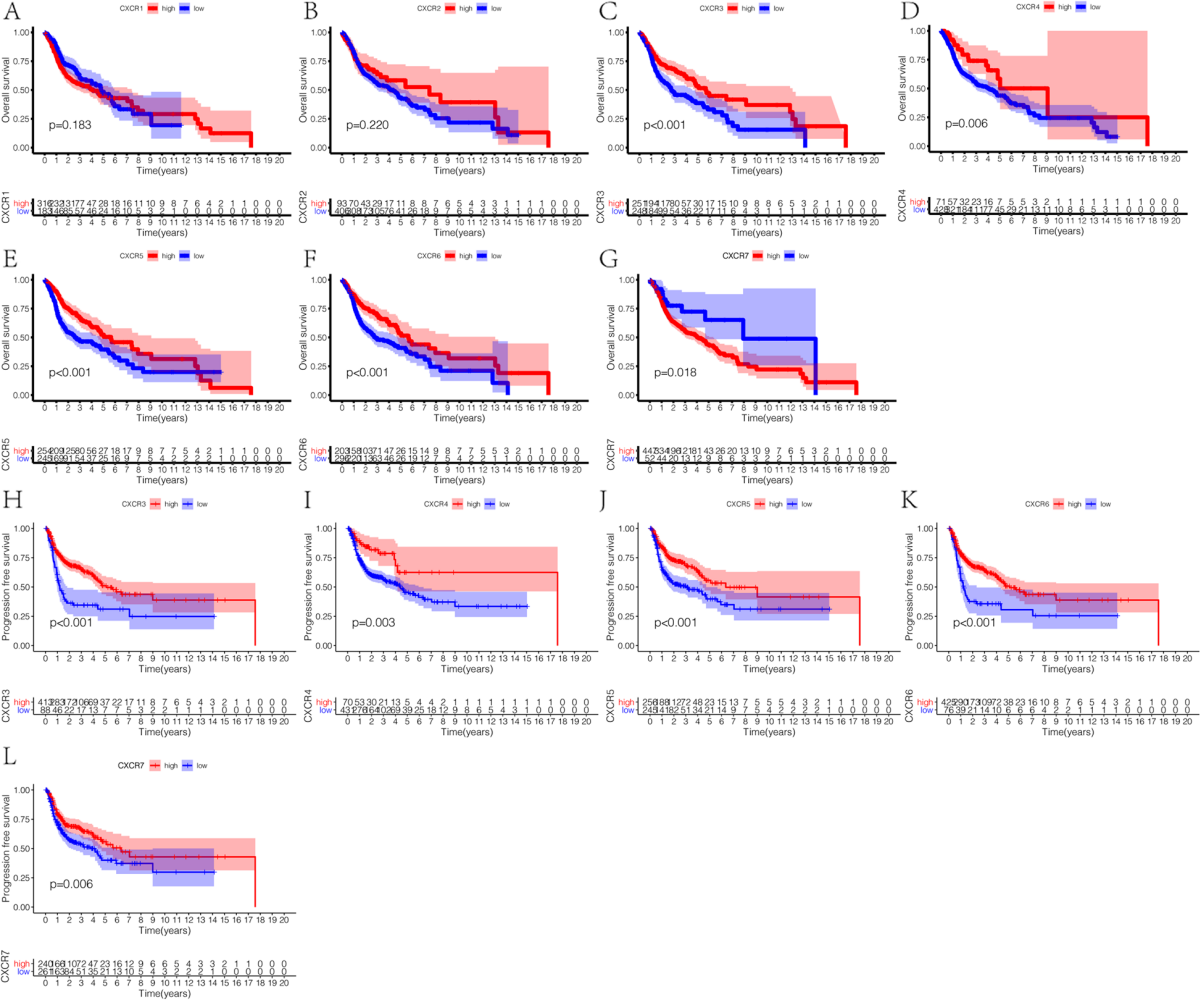
Prognostic value of *CXCRs* in HNSCC. **A**–**G** Survival outcomes and **H**–**L** recurrence outcomes

**Fig. 4:**
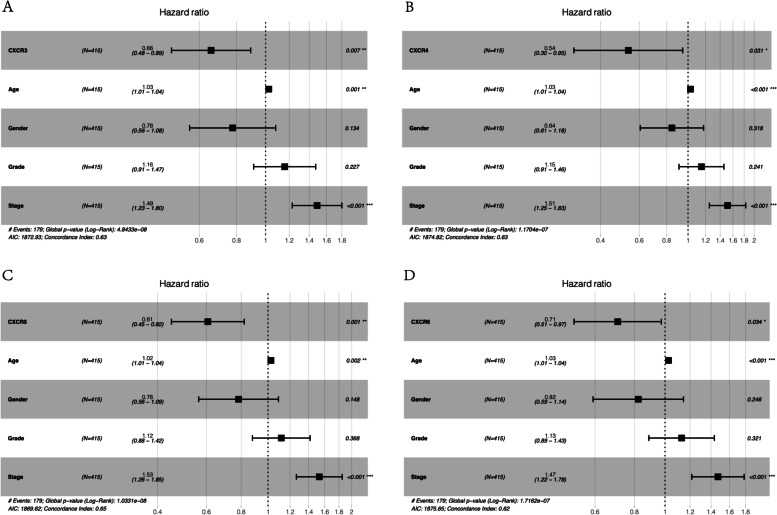
Results of multivariate Cox regression analyses of significant prognostic factors represented by forest plots. *p < 0.05. **p < 0.01. ***p < 0.001

To determine the correlation of *CXCR* family genes expression with the survival of HNSCC patients, how mRNA expression of *CXCRs* correlated with overall survivals on log-rank test was examined using Cutoff Finder (*CXCR1*: 0.255, *CXCR2*: 2.808, *CXCR3*: 1.205, *CXCR4*: 37.548, *CXCR5*: 0.0131, *CXCR6*: 1.390, *CXCR7*: 7.425). The overall survival and the progression-free survival in patients with *CXCR1-7* expression were plotted by utilizing Kaplan–Meier curves (Fig. 3). A significant association was observed between high mRNA expression of *CXCR3* to *CXCR6* and favorable overall survival (Fig. 3 C–F). In addition, databases (GSE41613 and GSE65858) were used for survival analysis to validate the survival value of *CXCR* family genes (Supplementary Fig. 1). Furthermore, there was a trend toward improved relapse-free survival with the high expression level of *CXCR3* to *CXCR6*, with statistical significance (log-rank test *p* < 0.01, Fig. 3 H–L). Subsequently, we aimed to determine the prognostic influences of *CXCR* members in HNSCC. To evaluate the predictive ability of differentially expressed *CXCRs* and their correlation with clinical characteristics, univariate Cox proportional hazards regression analysis was used. The association between the expression of four *CXCR* members (*CXCR3* to *CXCR6*) and the favorable outcome of HNSCC patients was found, so as the association of two clinical features (age and stage) with poor outcome (hazard ratio [HR] for *CXCR3*: 0.649; HR for *CXCR4*: 0.529; HR for *CXCR5*: 0.623; HR for *CXCR6*: 0.650; HR for age: 1.024; and HR for stage: 1.448) (Table 2). In the evaluation of the independent prognostic values of *CXCR3* to *CXCR6* along with the controlling of prognostic effects of these clinical features, the independent prognostic biomarkers of HNSCC outcome were reported by multivariate Cox proportional hazards regression analysis, i.e., the expression of *CXCR3* to *CXCR6* and two clinical parameters (age and stage) (Fig. 4).

**Table 2:** Univariate Cox proportional hazards regression analyses of *CXCR* members and clinical features in HNSCC

Parameter	Univariate analysis		
	Hazard ratio	95% ***CI***	***p***
Age	1.024	1.010–1.038	**0.001**
Gender	0.776	0.566–1.063	0.114
Grade	1.152	0.917–1.449	0.225
Stage	1.448	1.203–1.743	**9.15E-05**
*CXCR1*	1.208	0.883–1.652	0.238
*CXCR2*	0.809	0.546–1.199	0.290
*CXCR3*	0.649	0.481–0.877	**0.005**
*CXCR4*	0.529	0.301–0.931	**0.027**
*CXCR5*	0.623	0.462–0.839	**0.002**
*CXCR6*	0.650	0.474–0.892	**0.008**
*CXCR7*	1.208	0.921–2.216	0.111

Bold means *p* < 0.05

### Potential molecular mechanism underlying the roles of prognostic CXCRs in HNSCC

**Fig. 5:**
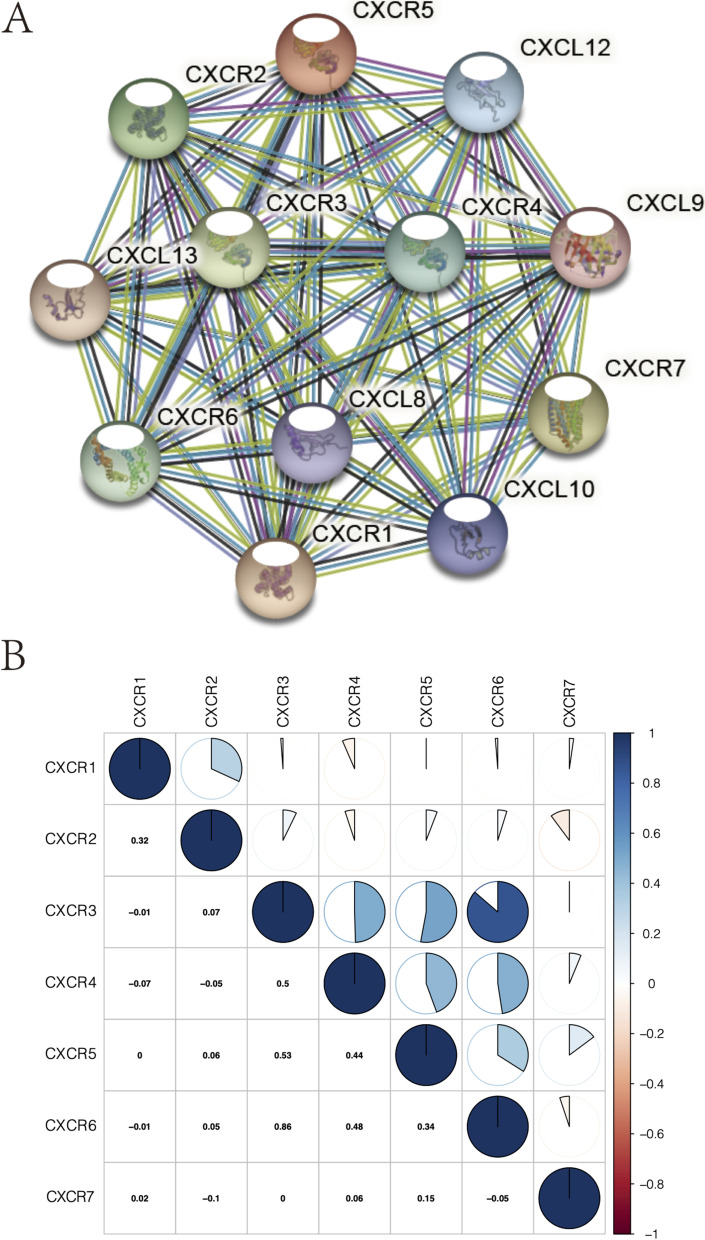
**A** PPI network among *CXCRs*. **B** Correlations between *CXCRs*

**Fig. 6:**
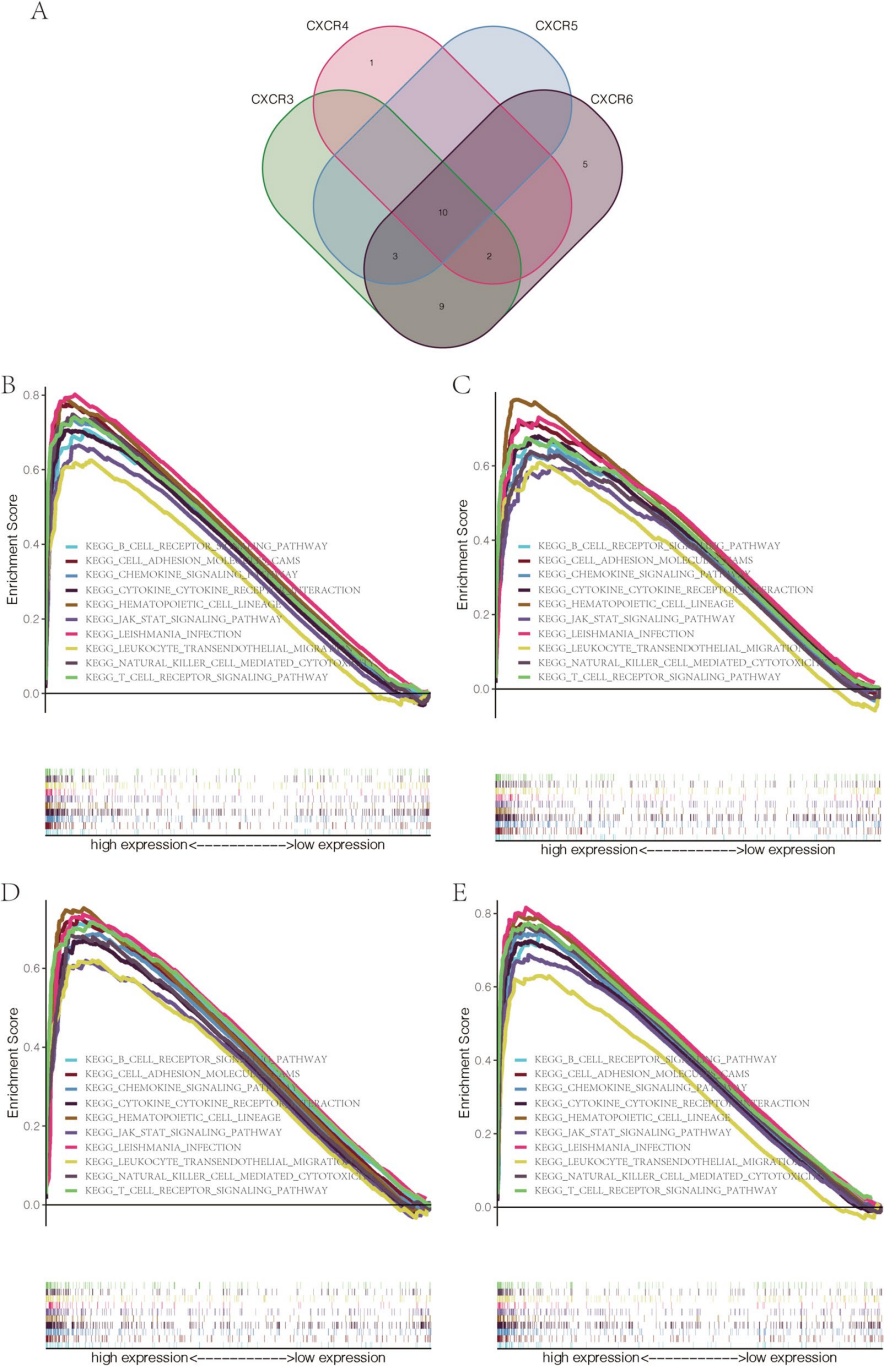
**A** Venn diagram showed *CXCR3-6* related to 10 common enriched pathways (KEGG). Cancer-related Kyoto Encyclopedia of Genes and Genomes (KEGG) pathways associated with **B***CXCR3*, **C***CXCR4*, **D***CXCR5*, and **E***CXCR6* based on a GSEA

On the basis of the gene expression data from TCGA, a PPI network was established by a Pearson correlation analysis combined with STRING database, aiming to determine the related protein interactions and possible correlation among *CXCR* family genes. A strong correlation was found between the *CXCRs* and interleukin-8 receptor activity (Fig. 5A), as well as the *CXCR1* gene and *CXCR2* (Fig. 5B). Besides, regarding the underlying biological mechanism of how the carcinogenesis of HNSCC is mediated by differential expression of *CXCR3* to *CXCR6*, a GSEA was carried out on the differentially expressed *CXCRs* with statistical prognostic value. Ten common cell signaling pathways closely related to high expression of *CXCR3* to *CXCR6* were proposed by the GSEA (Fig. 6A). Pathway analysis identified many immune-related pathways including a pathway involved in natural killer cell-mediated cytotoxicity, a process that is important in inflammation and immune responses (Fig. 6 B–E).

### Associations between TIICs and CXCRs in HNSCC

**Fig. 7:**
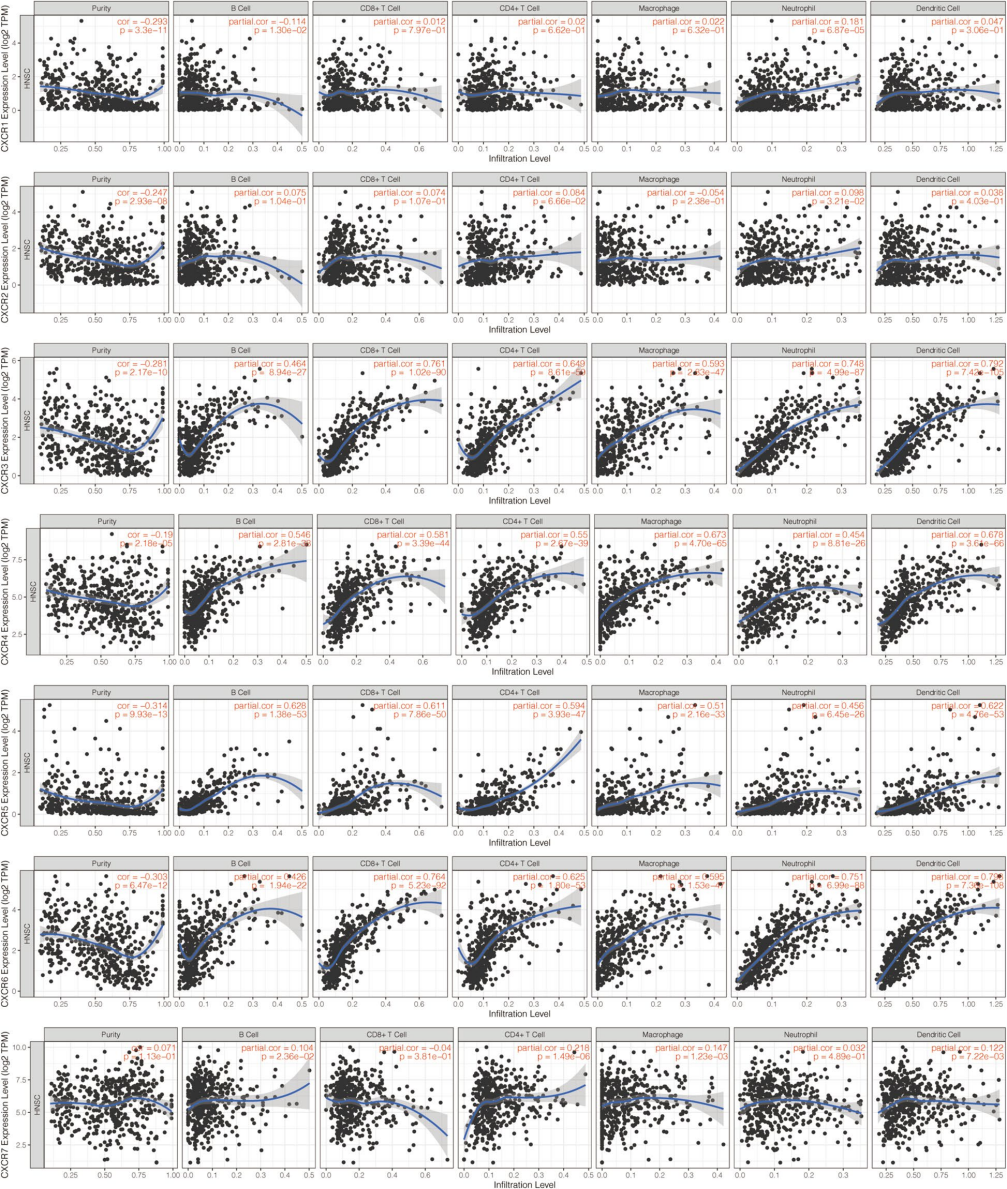
Correlations between tumor-infiltrating immune cells and independently prognostic *CXCRs* (*CXCR3*, *CXCR4*, *CXCR5*, and *CXCR6*)

**Fig. 8:**
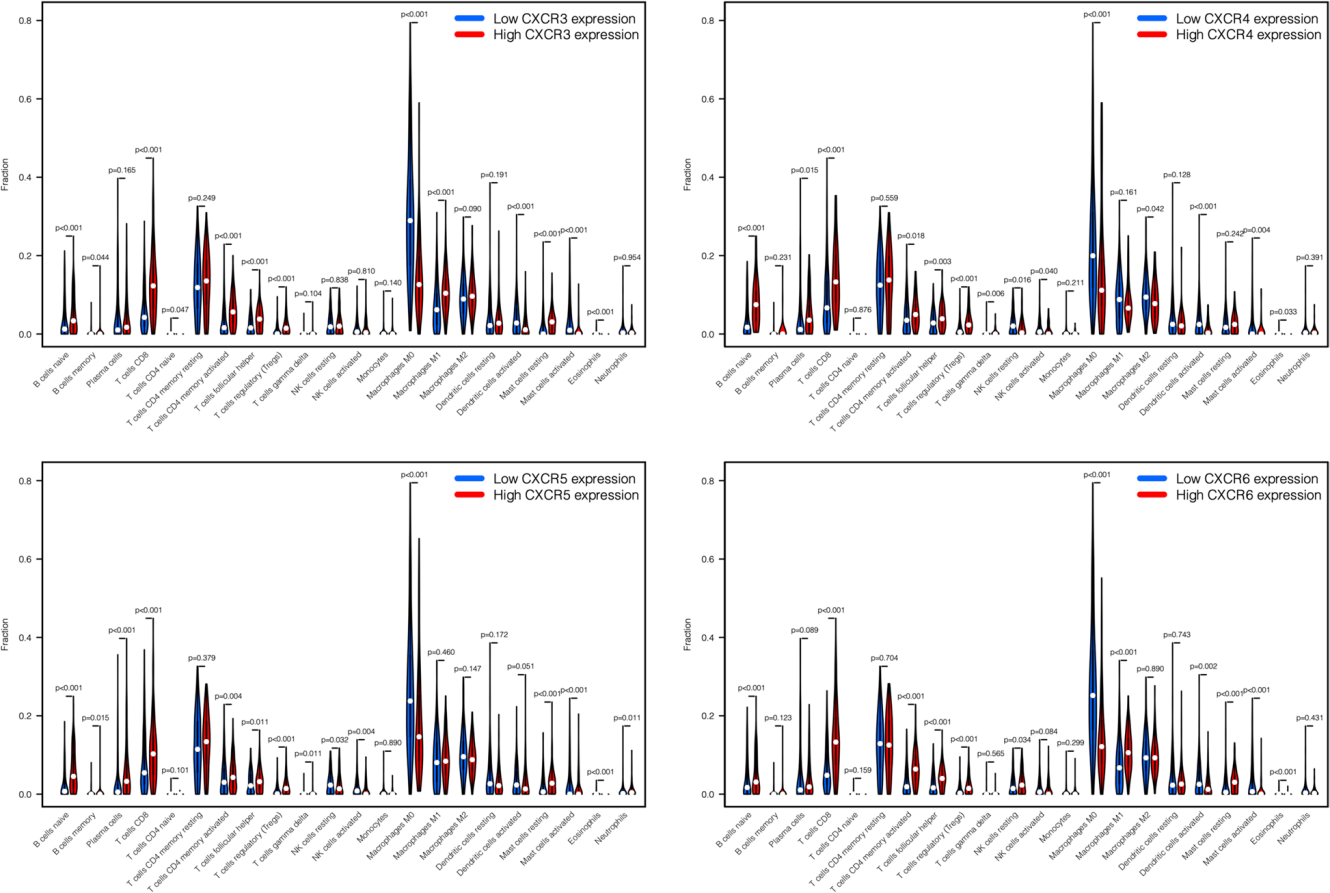
The abundance of tumor-infiltrating immune cells between high expression group and low expression group of *CXCR3*, *CXCR4*, *CXCR5*, and *CXCR6* in HNSCC

Given the growing evidence on the association between cancer prognosis and immunological features, the potential relevance of *CXCR* members in TIICs in HNSCC was further explored. For the evaluation of the association between immune cells surrounding tumor cells and certain gene products, we made use of the public resource provided by the TIMER database. The scatterplots were shown here presenting the expression of *CXCR* members against tumor purity. It was reported that in stromal-immune cells surrounding tumor cells, the high expression of *CXCRs* was anticipated to be negatively correlated with tumor purity but positively correlated in tumor cells. Consistent with the results mentioned above, *CXCR1* to *CXCR6* were highly expressed in HNSCC tissues, which showed a negative correlation with tumor purity (Fig. 7) and suggested that there was a significant correlation between TIICs and *CXCR* members, *CXCR3-6* in particular. The relationship between *CXCRs* gene expression and the abundance of 22 immune cell types in HNSCC was revealed by CIBERSORT results (Fig. 8). High *CXCR3* expression was significantly related with more regulatory T cells (Tregs), M0 macrophages, naive B cells, resting mast cells, follicular helper T cells, activated dendritic cells, activated mast cells, memory B cells, activated memory CD4+ T cells, M1 macrophages, CD8+ T cells, and less naive CD4+ T cells, and eosinophils infiltration. High *CXCR4* expression was associated with gamma delta T cells, more naive B cells, activated NK cells, Tregs, activated memory CD4+ T cells, M2 macrophages, follicular helper T cells, activated dendritic cells, and less plasma cells, CD8+ T cells, resting NK cells, M0 macrophages, activated mast cells, and eosinophils infiltration. High *CXCR5* expression was positively related with follicular helper T cells, naive B cells, Tregs, plasma cells, gamma delta T cells, CD8+ T cells, resting NK cells, resting mast cells, memory B cell, and negatively related with M0 macrophages, activated mast cells, eosinophils, activated memory CD4+ T cells, and activated NK cells. Moreover, it was also demonstrated that high *CXCR6* expression was positively related with activated memory CD4+ T cells, naive B cells, Tregs, CD8+ T cells, resting NK cells, follicular helper T cells, and negatively related with activated mast cells, M1 macrophages, resting mast cells, eosinophils, activated dendritic cells, and M0 macrophages. Taking these findings together, *CXCR* family members may play a crucial role in the regulation of the immune microenvironment in HNSCC, with the potential to make promising targets for the treatment of HNSCC patients.

## Discussion

CXC chemokine receptors represent a large subfamily of the G protein-coupled receptors superfamily. Chemokine receptors, as the name suggests, have the primary function of orchestrating cell trafficking, in particular the mobilization of immune cells to the sites of inflammation. Although the role of the *CXCR* gene family in the onset and progression of many human cancers has been highlighted by accumulating evidence, including breast cancer, bladder cancer, acute lymphoblastic leukemia (ALL), and acute myeloid leukemia (AML) [27–29], the exact prognostic values of *CXCRs* in HNSCC remain to be further explored. With increasing published or publicly available genomic data and a variety of online platforms, it is possible to investigate the expression profiles and clinical practice value of families of genes in human cancers. In the current study, the distinct expression profile, methylation, and prognostic values of *CXCRs* were clarified, as well as their involvement in various biological processes in HNSCC.

We carried out the evaluation of the *CXCRs* expression levels in different malignancies and found the high expression of *CXCR4* and *CXCR7* in HNSCC samples from ONCOMINE. And a previous study found that *CXCR4* was shown to be overexpressed in HNSCC tissues in comparison with the healthy tissues, with a fold change (FC) of 3.447 (*p*-value = 2.75E-13) [30]. Also, in another study, the mRNA level of *CXCR4* was found to have a 2.150-fold increase in HNSCC tissue (*p*-value = 4.68E-6) [25]. Moreover, a significantly high level of *CXCR7* mRNA expression was previously found in HNSCC tissues in other studies. For example, the FC of the *CXCR7* expression in HNSCC tissue was 2.185 (*p*-value = 3.24E-7) and 6.647 (*p*-value = 0.002), respectively, in the study by Matthew et al. and Gokce et al. [30]. The significant difference between the transcription level of *CXCR7* in HNSCC and that in healthy head and neck tissues was revealed in another study [31]. Moreover, TCGA data analysis implicated the high expression of *CXCR1/3/4/5/6/7* in HNSCC tissues compared to the healthy tissues. Although *CXCRs* have been confirmed to be highly expressed in various tumor tissues by previous research, this study is the first to present the expression of *CXCR1* to *CXCR7* in HNSCC. The methylation levels in promoter areas were further analyzed to explore the underlying mechanism of abnormal *CXCRs* expression in HNSCC. Genes are involved in apoptosis, cell proliferation, and cell cycle through gene silencing and reactivation, which can be caused by demethylation and methylation of cg sites in promoter regions. The regional context along with neighboring sites is concerned with methylation changes at individual cg site. According to Pearson’s correlation analysis, among the six differentially expressed *CXCR* members (*CXCR1*, *CXCR2* to *CXCR7*), the expression level of *CXCR4* is particularly affected by its methylation level, suggesting the possible crucial role of abnormal methylation in the aberrant expression of these genes. Yet, the abnormal expression of the *CXCR* gene family may also result from other epigenetic or genetic alterations like gene mutations and copy number changes.

No significant correlation of mRNA expression levels of *CXCR1* and *CXCR2* with OS and RFS was found in this study, while high expression of *CXCR3/4/5/6* turned out to be related to favorable survival outcomes in patients with HNSCC. Univariate and multivariate Cox analyses were carried out combined with parameters of age, stage, and the 7 identified hub genes, in the hope of establishing a relatively accurate prognostic model for patients with HNSCC. Our analysis data suggested the predictive potential of *CXCR* as four *CXCR* members (*CXCR3*, *CXCR4*, *CXCR5*, and *CXCR6*) were significantly related to better clinical outcomes in HNSCC, as reported in acute myeloid leukemia [32]. The underlying molecular mechanisms of abnormal expression of *CXCRs* regulating HNSCC carcinogenesis were further studied. The significant correlation among *CXCRs* and their vital role in the interaction with CXC motif chemokine 8/9/10/12/13 were clarified by Pearson’s correlation analysis and the PPI network. *CXCRs* expression was also concerned with some signaling pathways involved in cancer progression, as indicated by the GSEA, thus leading to the potential mechanism of CXCR carcinogenicity. The significant correlation between *CXCR3* to *CXCR6* and cytokine-cytokine receptor interaction as well as cell adhesion molecules (CAMs) was also worth noting, which indicated an interaction with CXC motif chemokine. The association between *CXCR* family genes and TIICs was observed by the TIMER and CIBERSORT analyses, offering favorable proof for the connection between *CXCRs* expression and the immune microenvironment in HNSCC. Taken together, our data supported the regulatory function of *CXCRs* family members in the HNSCC immune microenvironment, which deserves further research.

It is already known that *CXCR3* is primarily expressed on vascular cells, NK cells, tumor cells, and activated T lymphocytes and binds to *CXCL9* to *CXCL11* [10, 11]. CXCR3 has three different isoforms in humans, *CXCR3A*, *CXCR3B*, and *CXCR3Alt*, and its dual role in immune response and cancer has been reported. For example, in a previous study, *CXCR3* was found to be the key mediator of tumorigenesis and the main cause of poor response and early recurrence in multiple cancers in multiple cancers [33]. Conversely, another study reported that *CXCR3*-*CXCL9/10/11* signaling pathway contributed to the chemotactic movement of immune cells activated by *CXCR3* to the tumor site, thereby promoting the antitumor immune response [34]. The data presented here verified that *CXCR3* expression was positively associated with immune cell infiltration, suggesting the overexpression of *CXCR3* could probably increase the infiltration of immune cells of all sorts into the tumor microenvironment.

The crystal structures of *CXCR4* reveal the way chemokine receptors are regulated by various modulators binding to different or sometimes overlapping binding pockets. *CXCR4* has been implicated in the onset of a great number of tumors, due to the reason that this receptor is considered to be crucial in immune cells chemotaxis, tumor cell proliferation, invasion, metastasis, and angiogenesis [35]. Widely expressed in physiological conditions, *CXCR4* and its ligand *CXCL12* are essential regulators of hematopoiesis, cardiogenesis, and neurogenesis [36–39]. In addition, there were some strong CXCR4 inhibitors which as adjuvant treatments for anticancer therapies against human cancer [40, 41].

As a specific receptor of *CXCL13*, *CXCR5* mediates cancer functions regulated by *CXCL13* [42]. According to Singh et al., the expression of *CXCR5* was increased significantly only in squamous cell carcinoma (SCC) with lung node metastasis, but not in other subtypes of lung cancer [43]. Moreover, Ma et al. reported the contribution of CD4 + CXCR5 + T cells to antitumor immunity and its relation to better outcomes in non-small cell lung cancer [44].

*CXCR6* expression was discerned in different hepatoma cell lines with various metastatic properties. Also, CXCR6 contributes to the microenvironment by inducing inflammatory cytokines, leading to the invasion and metastasis of hepatoma cells [45]. The high expression of *CXCR6* in HNSCC was revealed by TCGA database analysis in this study, especially in HNSCC patients at the advanced stage or the elder. Furthermore, high expression of *CXCR6* was proven to be connected to improve OS in HNSCC, and meanwhile, 5 CpGs of *CXCR6* make a difference to favorable survival. *CXCR6*-*CXCL16* axis also recruits immune cells to cancer sites just like the way of other *CXCRs*, thereby affecting the progression of cancer. However, the cell-specific functions of *CXCR6* remain unclear to a large extent [46]. Currently, there were no known drugs that specifically target *CXCR5* and *CXCR6*.

Overall, this study performed a comprehensive and thorough analysis of the expression and prognostic values of the *CXCRs* in HNSCC using publicly available TCGA databases. This study found that some of the *CXCR* family members could serve as clinical biomarkers of HNSCC. Our findings highlighted the vital function of *CXCR3* to *CXCR6* in HNSCC progression, as well as their clinical significance in HNSCC. Furthermore, we provided an insight into the role of *CXCR3* to *CXCR6* in mediating different signaling pathways in HNSCC. Our study also suggested that *CXCR3-6* mediated inhibition of HNSCC progression may correlate with T-cell receptor signaling pathway, leukocyte trans-endothelial migration, and natural killer cell cytotoxicity. Nevertheless, the specific role of *CXCR3* to *CXCR6* in HNSCC requires future exploration.

## Conclusions

In general, despite the promising results achieved, there are still some limitations in the present study. We performed all analyses and obtained the results on the sole basis of data from the TCGA, without verifying the expression levels of *CXCRs* protein and mRNA in HNSCC as well. Nevertheless, as suggested by our findings, targeting *CXCR* family members could be a promising therapeutic approach for treating patients with HNSCC.

## Supplementary Information

**Additional file 1: Supplementary Figure 1**. The survival value of *CXCR* family genes in GSE41613 and GSE65858.

**Additional file 2: Supplementary Table 1**. Clinical characteristics of the patients from multiple institutions.

## Data Availability

The gene expression data and clinical features used for bioinformatics analyses in this study are publicly available on the Cancer Genome Atlas (TCGA) program website (https://portal.gdc.cancer.gov). The authors are solely responsible for interpreting and reporting these data.

## Abbreviations

HNSCC: Head and neck squamous carcinoma
*CXCRs*: CXC chemokine receptor gene family members
GSEA: Gene set enrichment analysis
*CXCL*: CXC motif chemokine ligand
NK cells: Natural killer cells
TCGA: The Cancer Genome Atlas
HTSeq: High-throughput sequencing
FPKM: Fragments per kilobase of transcript per million mapped reads
GDC: Genomic Data Commons
PFS: Progression-free survival
OS: Overall survival
PPI: Pearson correlation and protein-protein interaction
FDR: False discovery rate
TIICs: Corrections between tumor immune-infiltrating cells
FC: Fold change
ALL: Acute lymphoblastic leukemia
AML: Acute myeloid leukemia
Tregs: Regulatory T cells
