# Supplementary figures and images for: Expression profile and prognostic value of CXCR family members in head and neck squamous cell carcinoma
Source: World J Surg Oncol. 2022 Aug 17;20:259. doi: 10.1186/s12957-022-02713-z (PMC9382762; doi:10.1186/s12957-022-02713-z)

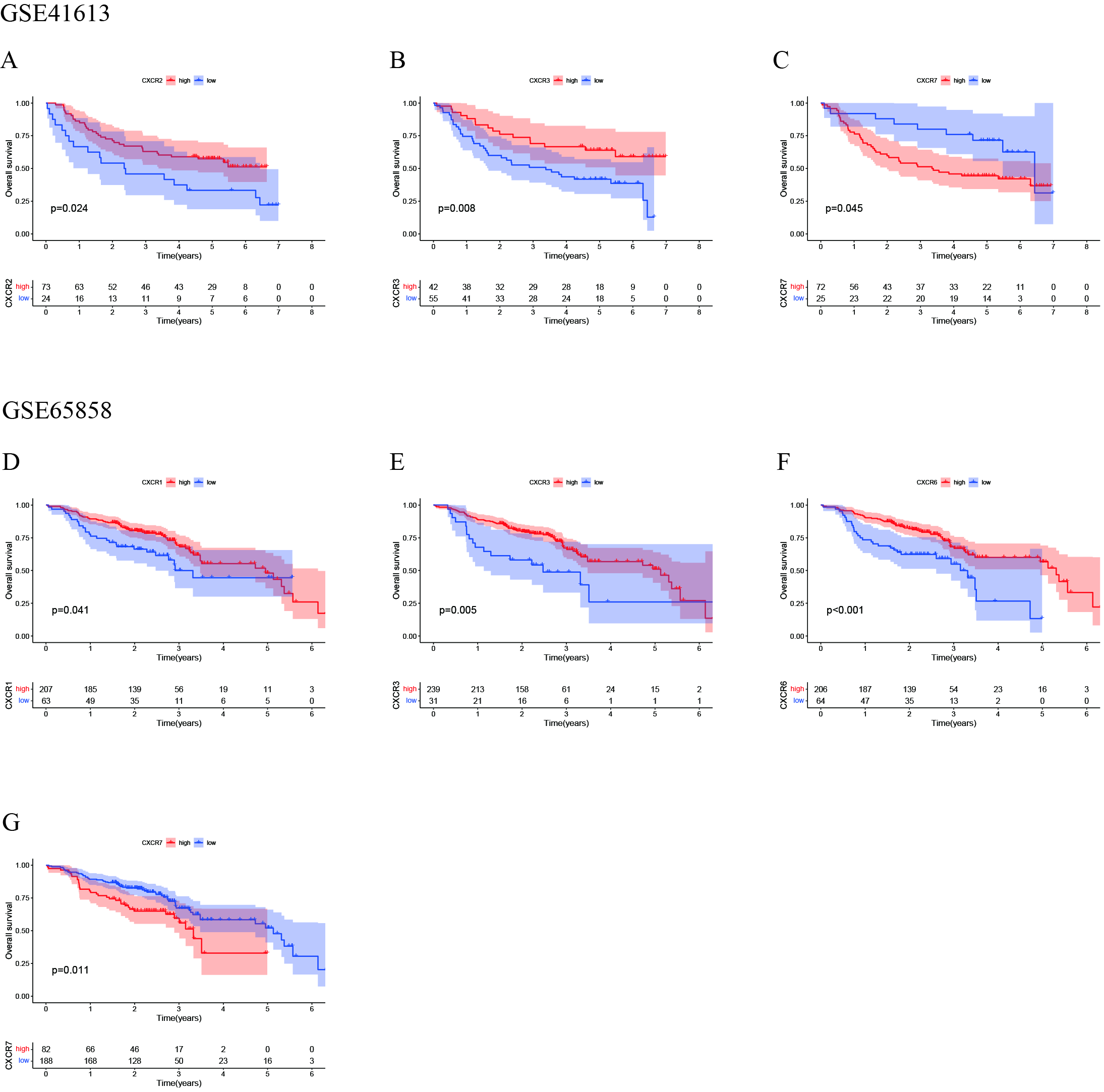

Supplement: Supplementary file 1 — Additional file 1: Supplementary Figure 1. The survival value of CXCR family genes in GSE41613 and GSE65858. [file 12957_2022_2713_MOESM1_ESM.tiff]
