# Supplementary material for: Expression profile and prognostic value of CXCR family members in head and neck squamous cell carcinoma
Source: World J Surg Oncol. 2022 Aug 17;20:259. doi: 10.1186/s12957-022-02713-z (PMC9382762; doi:10.1186/s12957-022-02713-z)
Supplement: Supplementary file 2 — Additional file 2: Supplementary Table 1. Clinical characteristics of the patients from multiple institutions. [file 12957_2022_2713_MOESM2_ESM.docx]

**Supplementary Table 1.** Clinical characteristics of the patients from multiple institutions

| Characteristics | TCGA n=527 | GSE41613 n=97 | GSE65858 n=270 |
| --- | --- | --- | --- |
| Age (years) |  |  |  |
| ≤60 | 260 | 50 | 153 |
| >60 | 266 | 47 | 117 |
| Gender |  |  |  |
| Male | 385 | 66 | 223 |
| Female | 142 | 31 | 47 |
| Grade |  |  |  |
| G1 | 63 | — | — |
| G2 | 310 | — | — |
| G3 | 125 | — | — |
| G4 | 7 | — | — |
| GX | 18 | — | — |
| Unknow | 4 | — | — |
| Stage |  |  |  |
| Stage I+II | 100 | 41 | 55 |
| Stage III+IV | 352 | 56 | 215 |
| Unknow | 75 | 0 | 0 |
| OS state |  |  |  |
| Alive | 305 | 46 | 94 |
| Death | 223 | 51 | 176 |
